# Isoforms of Base Excision Repair Enzymes Produced by Alternative Splicing

**DOI:** 10.3390/ijms20133279

**Published:** 2019-07-03

**Authors:** Elizaveta O. Boldinova, Rafil F. Khairullin, Alena V. Makarova, Dmitry O. Zharkov

**Affiliations:** 1 RAS Institute of Molecular Genetics, 2 Kurchatova Sq., 123182 Moscow, Russia; 2 Institute of Fundamental Medicine and Biology, Kazan (Volga Region) Federal University, 9 Parizhskoy Kommuny Str., 420012 Kazan, Russia; 3 Novosibirsk State University, 1 Pirogova St., 630090 Novosibirsk, Russia; 4 SB RAS Institute of Chemical Biology and Fundamental Medicine, 8 Lavrentieva Ave., 630090 Novosibirsk, Russia

**Keywords:** alternative splicing, DNA glycosylases, apurinic/apyrimidinic endonuclease, DNA polymerase beta

## Abstract

Transcripts of many enzymes involved in base excision repair (BER) undergo extensive alternative splicing, but functions of the corresponding alternative splice variants remain largely unexplored. In this review, we cover the studies describing the common alternatively spliced isoforms and disease-associated variants of DNA glycosylases, AP-endonuclease 1, and DNA polymerase beta. We also discuss the roles of alternative splicing in the regulation of their expression, catalytic activities, and intracellular transport.

## 1. Introduction

Base excision repair (BER) is the predominant and conserved pathway that corrects small DNA lesions derived from oxidation, deamination, and alkylation (reviewed in [1,2,3,4,5]). BER is initiated by a DNA glycosylase that removes damaged or mismatched nucleobase, leaving an apurinic/apyrimidinic site (AP site). At least 11 human DNA glycosylases are known, each recognizing one or a few related lesions, but also demonstrating overlapping specificities. AP sites are further cleaved by an AP endonuclease (APEX1 in humans) yielding a 3′ hydroxyl and a 5′ deoxyribose phosphate moiety (dRP). Alternatively, bifunctional DNA glycosylases not only excise damaged bases but follow with damaged-strand nicking by β-elimination. Regardless of the nick origin, it can then be processed by either short-patch (where a single nucleotide is replaced) or long-patch (where 2 to 13 nucleotides are replaced) BER. In the former case, after the AP site cleavage, DNA repair polymerase (DNA polymerase beta, or Pol β, in human cells) removes the dRP group, through its dRP lyase activity, and fills the gap. In the latter sub-pathway, Pol β and possibly other DNA polymerases displace the nicked strand in the 5′→3′ direction, and the resulting flap is then excised by FEN1 endonuclease. BER plays a crucial role in maintaining genomic stability (recently reviewed in [6]). A number of mutations and single-nucleotide polymorphisms were shown to be associated with an increased risk of human diseases including cancer (reviewed in [7,8,9]).

Alternative splicing of pre-mRNA has been shown to affect about 95% of human genes [10,11,12], and is involved in the regulation of normal physiological functions as well as in pathologic processes [13,14]. Alternative splicing increases functional diversity and provides additional regulatory opportunities. In particular, protein isoforms generated by alternative splicing could be different in their catalytic capacity, subcellular localization, or protein–protein interactions. In addition, alternative splicing could negatively regulate gene expression (e.g., regulated unproductive splicing and translation realized through the nonsense-mediated decay of alternatively spliced mRNA isoforms harboring a premature termination codon) [15].

Many BER enzymes are associated with several mRNA and protein variants from a single gene. The transcript diversity of BER enzymes resulting from alternative splicing has yet to be explored. Little is known about the effect of common alternative splicing events on the BER enzymes activities and functions. In this review, we address alternative splicing of key BER enzymes: DNA glycosylases, APEX1, and Pol β. We summarize the studies describing common alternatively spliced isoforms of BER enzymes and their known disease-associated variants. We also discuss the possible roles of alternative splicing in the regulation of BER enzyme expression and activities.

## 2. Isoforms of DNA Glycosylases and Apurinic/Apyrimidinic Endonuclease

### 2.1. Uracil–DNA Glycosylase (UNG)

**Figure 1:**
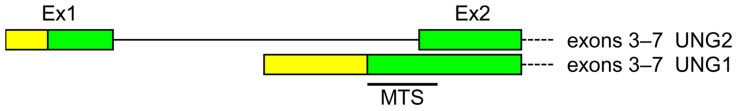
Organization of the alternative transcription initiation in the human *UNG* gene. Untranslated regions are yellow, coding sequences are green. MTS—mitochondrial targeting sequence.

Uracil–DNA glycosylase was the first discovered human DNA glycosylase and the first one for which the existence of different mRNA and protein isoforms was confirmed [16,17,18,19,20,21,22,23]. The *UNG* gene contains seven exons and two alternative transcription initiation sites that generate two mRNA and two protein isoforms (often called UNG1 and UNG2). Exon 1 is only included in the *UNG2* mRNA isoform, while exon 2 is the first exon of *UNG1* and incorporates part of intron 1 where the *UNG1* transcription start is located [19,20,23,24,25] (Figure 1). Direct promoter activity mapping suggests that there might be yet another untranslated exon upstream of exon 1, important for cell cycle-dependent regulation involving the E2F family transcription factors [26,27].

Both UNG isoforms share the 269 amino acids (aa) long catalytic domain and possess nearly identical enzymatic activity, removing uracil from any context in DNA [28,29,30]. The isoform-specific functions entailed by the unique N-termini (35 aa in UNG1, 44 aa in UNG2) are related primarily to intracellular trafficking, protein–protein interactions, and regulatory post-translational modification.

The UNG1 protein isoform carries a classical N-terminal mitochondrial targeting sequence (MTS) and is imported into mitochondria, whereas the UNG2 isoform is localized to the nucleus due to a complex nuclear localization signal (NLS) split between its unique N-terminus and the catalytic domain common with UNG1 [19,23,31]. Interestingly, while the mouse *Ung* gene has a similar structure and also produces two protein isoforms, mouse UNG1 protein has an N-terminus quite different from that of human UNG1 and is sorted both to mitochondria and to nuclei [32,33].

Under oxidative stress, UNG1 forms a disulfide bond through its Cys5 with a major mitochondrial antioxidant protein peroxiredoxin 3, which protects UNG1 from damage and reduces mitochondrial DNA oxidation [34]. The nuclear isoform, UNG2, is cell-cycle regulated, targeted for degradation in the late S phase through ubiquitylation dependent on phosphorylation within the isoform-specific N-terminal region [35,36,37]. On the other hand, UNG2 is phosphorylated at Thr6, and its dephosphorylation by protein phosphatase 1D partially suppresses the enzyme activity [38]. In addition, the N-terminal tail of UNG2 interacts with proliferating cell nuclear antigen (PCNA) clamp and replication protein A (RPA) [39], raising the possibility of replication-coupled uracil repair, which makes sense considering massive amounts of uracil misincorporated into DNA during the replication [40].

### 2.2. Thymine–DNA Glycosylase (TDG)

TDG, which removes an array of pyrimidine oxidation/deamination products, is now considered to be a major component of the epigenetic active cytosine demethylation system rather than a bona fide DNA repair enzyme [41,42]. Human *TDG* gene contains ten exons and generates two mRNA isoforms, one of which produces a full-length, extensively characterized protein, whereas the second encodes a truncated protein lacking part of the active site and is likely non-functional [24,43,44,45,46]. The mouse *Tdg* gene expresses two mRNAs with different incorporation of exon 1 parts that have different translation initiation sites [47,48,49,50,51]. The polypeptides, TDGa and TDGb, appear to behave identically with respect to their ability to bind SUMO-1 but were not compared otherwise [51].

### 2.3. Single-Strand-Selective Monofunctional Uracil-DNA Glycosylase 1 (SMUG1)

SMUG1 has enzymatic activity and substrate specificity similar to UNG and is regarded as a backup for UNG [30,52]. Its gene contains six exons, with multiple transcription start sites, cryptic splicing sites within exons 1 and 6, and skipping of exons 2–5 producing 32 known mRNA isoforms, the largest number among all DNA glycosylases [24,44,53,54,55,56] (Table 1). However, they encode only five different protein isoforms, of which only one has been biochemically characterized [52,54], whereas all the rest are predicted to lack important parts of the catalytic domain.

### 2.4. Methyl-Binding Domain-Containing Protein 4 (MBD4)

MBD4 is an enzyme that removes cytosine and 5-methylcytosine deamination products from CpG dinucleotides and has a methyl-binding domain directing it to CpG-islands; it may have both DNA repair and epigenetic demethylation functions [41,58,59]. The human *MBD4* gene possesses eight exons; skipping of exon 3 and alternative splicing donor sites in exon 7 produce five mRNA and five protein isoforms [44,58,59,60,61,62,63]. Two protein isoforms (3 and 4) terminate prematurely and lack portions of the catalytic domain. In addition to the major full-length protein isoform 1, a splice variant (isoform 5) skipping exons that code for the methyl-binding domain but possessing an intact glycosylase domain have been characterized [63]. The protein displayed robust uracil-excising activity, coincident with the truncated MBD4 used in many biochemical studies [64,65,66]. Two probable pathogenic splice variant mutations of *MBD4* associated with glioblastoma and uveal melanoma [67] were reported in the Human Gene Mutation Database (HGMD) [68] (Table 2).

### 2.5. Endonuclease III-Like Protein (NTHL1)

NTHL1 is the main human glycosylase for the repair of oxidized pyrimidines [74,75]. The contains six exons, with alternative transcription start sites in exons 1 and 2, and exon 3 skipped in one of three known mRNA variants [25,44,74,75,76,77,78,79]. Only one of three resulting protein isoforms has intact catalytic domain and can be considered functional. The translation initiation site of the *NTHL1* open reading frame is ambiguous, since the first 16 sequence positions contain three methionines and four predicted mRNA capping sites. However, the enhanced green fluorescent protein (EGFP)-tagged NTHL1 starting at any of these three Met positions are localized identically, both to nucleus and to mitochondria [79,80,81]. Remarkably, an abnormal splicing-causing mutation in intron 4 of the *NTHL1* gene associated with several tumor phenotypes (colonic adenocarcinoma, bladder carcinoma, intradermal nevi, meningioma, multiple seborrheic keratoses, basal cell carcinoma, multiple colorectal adenomas, squamous cell carcinoma, and invasive ductal carcinoma) was reported (Table 2).

### 2.6. 8-Oxoguanine–DNA Glycosylase (OGG1)

**Figure 2:**
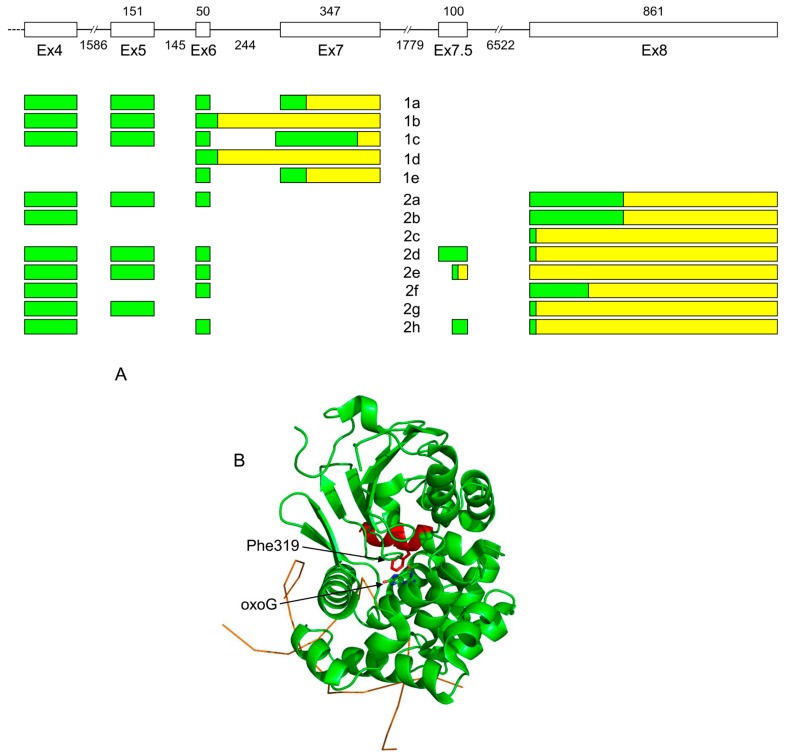
(**A**) Organization of exons 4–8 and alternative splicing products of the human *OGG1* gene. (**B**) Structure of OGG1-1a protein (PDB ID 1EBM, [98]). DNA backbone is shown schematically as an orange line. The protein part encoded by exon 7 and thus missing in OGG1-1b and all group 2 isoforms are colored red. Phe319 forms a wall of the enzyme active site and stacks against the oxoG base; these interactions have to be remodeled in OGG1 group 2. Untranslated regions are yellow, coding sequences are green.

OGG1 is responsible for removal of an abundant pre-mutagenic oxidative lesion, 8-oxoguanine (oxoG). OGG1 together with another DNA glycosylase, MUTYH, and 8-oxodGTPase NUDT1 (MTH1) constitute a so-called GO system that controls oxoG at pre- and post-replicative levels [82,83]. The human *OGG1* gene contains eight exons plus one small, irregularly-used exon 7.5 [84,85,86]. The intron between exons 7 and 8 contains nearly a half of calmodulin kinase 1 (*CAMK1*) gene transcribed in the opposite direction. Alternative splicing generates two groups of *OGG1* mRNA isoforms. *OGG1* group 1 isoforms (1a–1e) include exons 1 to 7, whereas exon 8 substitutes for exon 7 in group 2 mRNAs (2a–2h); within those groups, isoforms differ from each other by the use of some internal exons and alternative splice sites [44,84,85,87,88,89,90,91,92,93,94,95,96,97] (Figure 2A). Interestingly, group 2 isoforms have been reported in primates only, and even extensively-annotated transcriptomes of other animals, such as mice, chicken, or zebrafish, show no signs of them.

Following the mRNA differences, the protein isoforms of OGG1 fall in two groups with different C-termini. Since group 2 protein isoforms contains only the N-terminal MTS, while group 1 isoforms also contains the NLS in its C-terminal part, it was initially believed that these groups comprise mitochondrial and nuclear proteins, respectively [95], and groups 1 and 2 are still often referred to as nuclear and mitochondrial OGG1 isoforms, respectively. However, immunocytochemical localization of OGG1-1a detected it both in the nucleus and in the cytoplasm in a speckled pattern characteristic of mitochondrial proteins, whereas OGG1-1b, -1c, and -2a were excluded from the nucleus and found only in mitochondria [80,99]. Moreover, high-resolution microscopy indicates that OGG1-1a resides in the mitochondrial matrix, associated with mtDNA in the nucleoid [99]. The N-terminal target peptide is required for mitochondrial localization of OGG1 regardless of the presence of the NLS, and is necessary to complement the mitochondrial function under the oxidative stress conditions [80,99]. The G12E somatic mutation found in renal clear cell carcinomas abolishes mitochondrial localization of OGG1-2a without affecting the activity of OGG1-1a [100].

OGG1-1a protein has been extensively biochemically characterized. After the structure of OGG1-1a was determined [98], it became evident that its active site is partly built from the polypeptide region unique for this isoform, including Phe319 that stacks against oxoG in the recognition pocket (Figure 2B). Therefore, other isoforms, if active, have to use some other mode of oxoG recognition. In line with these structural considerations, the recombinant OGG1-2a protein, which deviates from OGG1-1a starting at position 317, was reported to lack enzymatic activity [101]. Paradoxically, OGG1-1b, which also lacks Phe319, was reported to possess oxoG-excising activity at the level comparable with OGG1-1a [102]. Overall, the question of enzymatic activity of various OGG1 protein isoforms remains unsolved.

Despite the lack of activity of OGG1-2a isoform, the oxoG excision activity encoded by *OGG1* is found in mitochondria [103,104]. In principle, participation of OGG1-2a still cannot be excluded, since the protein expressed intracellularly in *Escherichia coli* might not fully reflect the properties of the protein after its unfolding and refolding by the mitochondrial import system [105,106]. However, given that group 1 isoforms are also detected in mitochondria by Western blotting and immunocytochemistry [80,99,101], it is very likely that OGG1-1a is responsible for the oxoG removal activity there, and group 2 isoforms may play some other role. Intriguingly, OGG1-1a targeted to mitochondria by additional SOD2 signal peptide makes cells more resistant to oxidative stress even if OGG1 carries inactivating mutations in the active site; this ability apparently depends on blocking oxidant-induced decreases in mitochondrial aconitase activity [107].

In the yeast two-hybrid system, OGG1-2a interacts with NADH:ubiquinone oxidoreductase subunit B10, an integral part of the NADH dehydrogenase (ubiquinone) complex residing in the mitochondrial inner membrane [108]. Selective knockdown of both group 1 and group 2 by targeting exons 7 and 8, respectively, sensitizes cells to oxidative stress, impairs the respiration, and increases the oxoG content in their mitochondrial DNA, while overexpression of OGG1-2a but not of OGG1-1a increases oxoG removal activity in mitochondria and protects cells from oxidative damage [108,109].

It is not clear yet whether the minor mRNA isoforms (1b–1e, 2b–2h) are translated. Western blotting with monoclonal antibodies directed against the putative NLS reveals several immunoreactive OGG1 protein bands [96,110], suggesting that some nuclear isoforms do appear in human cells. Cells under oxidative stress have been reported to accumulate shorter OGG1 polypeptides that are either minor isoforms or produced by apoptosis-related proteolysis [111,112].

Some pathologic processes can change the balance of OGG1 isoforms, although no cause–effect relations have been established. Group 2 isoforms are increased in the substantia nigra in Parkinson’s disease patients [113]. *OGG1-1a* and *-1c* transcripts are significantly upregulated, whereas *OGG1-1b* is downregulated in Alzheimer’s disease patients in comparison with age-matched controls [114]. 

The HGMD contains three splice-site altered *OGG1* variants. One of them is reported as a disease-causing variant (colorectal cancer) and two mutations are marked as disease-associated polymorphisms associated with breast cancer (Table 2) [70,71]. A possibly pathogenic mutation *OGG1* c.137G>A affecting the last nucleotide in exon 1 was discovered in heterozygote in a colorectal cancer patient who also carried a heterozygous I223V mutation in the coding region of *MUTYH* (Table 2). The c.137G>A variant led to a complete disappearance of mutant mRNA from the patients’ cells, with no aberrant splicing products present [70,115].

### 2.7. MutY Homolog (MUTYH)

MUTYH is responsible for removing A mispaired with oxoG, and also can excise several oxidized purine derivatives [116,117]. The *MUTYH* gene consists of 17 exons [25,44,45,56,118,119,120,121]. Alternative transcription initiation and splicing of the *MUTYH* pre-mRNA produces at least 13 mRNA isoforms and nine protein isoforms with different 5′-terminal mRNA and N-terminal protein sequences [120,122]. The experimentally identified transcripts form three groups—α, β, and γ—with different transcription initiation sites, and there are multiple instances of exon skipping and alternative splice site use [120]. One source of mRNA variability may be the overlap between exon 1 of *MUTYH* and exon 1 of *TOE1* gene transcribed in the opposite direction under the control of early growth response protein 1 (EGR-1) promoter [55,123]. The major nuclear protein isoform, MUTYHα1, is 546 aa long. The functions of other protein isoforms are unclear. All but one have an intact catalytic domain and are likely to be functional; they may have different catalytic activity and vary in the opposite-base specificity [124], and some of them are probably mitochondrial [80,119,120]. The N-terminal 32-aa peptide also contains a binding site for replication protein A, implying that some isoforms of MUTYH may participate in replication-coupled repair [125]. In mouse cells, three mRNA isoforms are synthesized, two encoding identical polypeptides (MUTYHα) homologous to the major human isoform, while the third uses an alternative translation initiation site and skips one internal exon, resulting in a protein lacking the DNA minor groove-binding motif and likely inactive [122]. In the rat brain, specific mitochondrial isoforms of MUTYH protein have been observed, which are developmentally regulated and induced by respiratory hypoxia in the hippocampus [126,127].

The involvement of MUTYH deficiency in the pathogenesis of colorectal cancer [116,128] spurred interest in possible splice-site mutations in human malignancies. A germline variant, c.892-2A>G, encoding a truncated protein without the NUDIX domain, was discovered in gastric cancer patients; however, its association with cancer risk has not been established due to a small number of cases investigated [129]. Another polymorphism discovered in VMRC-LCD lung cancer cell line, IVS1+5G/C, is located in the first intron and causes incorporation of extra 237 nucleotides in the 5′-UTR of group β isoforms [121]. While it does not change the protein sequence, it appears to reduce the translation efficiency of the carrier mRNAs [121].

The search of *MUTYH* pathogenic splice variants in the HGMD revealed 19 splice-site mutations reported as a disease-causing mutation, eight splice mutations marked as probable pathological mutation, and one mutation caused reduced translation efficiency of *MUTYH* transcripts. The majority of *MUTYH* splice mutations are implicated in MUTYH-associated polyposis and other types of colorectal cancer (Table 3).

### 2.8. Methylpurine–DNA Glycosylase (MPG)

MPG (alias AAG or APNG) excises ring-alkylated purines and several other purine-derived lesions, such as etheno adducts and hypoxanthine [143]. The *MPG* gene contains six exons that produce three mRNAs and three protein isoforms (MPGa–MPGc) due to alternative transcription initiation and alternative splicing that produces mRNA with one of two alternative first exons [144,145,146]. The protein isoforms are different only in a short N-terminal sequence and are 283–298 aa long [144,145]. All isoforms appear to be ubiquitously expressed, and the respective proteins show identical activity, substrate specificity, and the ability to protect *E. coli* from alkylation DNA damage when produced ectopically [144,147,148].

### 2.9. Endonuclease VIII-Like Proteins (NEIL1, NEIL2, and NEIL3)

NEIL1, NEIL2, and NEIL3 are homologs of bacterial endonuclease VIII (Nei); they are involved in the repair of oxidized bases and most likely are needed in special cases, such as repair in non-canonical DNA structures, transcription-coupled repair, or repair in certain cell types or at specific developmental stages [149,150,151]. In humans, *NEIL1* gene contains eleven introns, generates four alternatively spliced mRNAs, and encodes four protein isoforms [24,44,55,152,153]. Of these, only one contains no deletions of the essential parts of the protein; the others have not been studied. In mice, in addition to mRNA encoding full-length NEIL1, two splice variants were detected, one containing full intron 4, another including the first 10 nt of intron 1. Both variants also produce truncated proteins, which lack fully or partially the C-terminal DNA-binding domain and possess no catalytic activity [154].

Interestingly, *Arabidopsis* MMH-1 protein, belonging to a group of plant and fungal H2TH glycosylases that is phylogenetically closest to NEIL1, has multiple isoforms with variable C-termini due to alternative splicing of its pre-mRNA [155,156,157]. Only two variants, MMH-1 and MMH-2, have been characterized biochemically, and only the former had the glycosylase and AP lyase activities, whereas the latter lacks part of the C-terminal domain and is inactive [156].

The *NEIL2* gene contains five exons and produces eight mRNA isoforms due to alternative splicing [24,44,53,55,153,158,159]. Three protein isoforms are produced; two of them lack parts of the catalytic domain and are presumably inactive.

The *NEIL3* gene contains ten exons. A single mRNA and a single protein isoform are known [44,45,55,62,153,158,160].

One splice variant of NEIL1 reported as disease-associated polymorphism with additional functional evidence was identified but its clinical significance is not obvious [72]. NEIL2 has one splice-site mutation reported as probable pathological splice variant mutation associated with multiple colorectal adenomas [73] (Table 2).

### 2.10. Apurinic/Apyrimidinic Endonuclease (APEX1)

APEX1 (also known as APE1, HAP1, or Ref-1) catalyzes the next step after DNA glycosylases, hydrolyzing the DNA backbone 5′ to the AP site formed by the DNA glycosylase action [161]. The *APEX1* gene contains five exons and produces four mRNA isoforms that differ by using splice donor sites in the untranslated exon 1 and all produce the same polypeptide [44,162,163,164,165,166,167,168,169,170,171,172].

## 3. Isoforms of DNA Polymerase β

Pol β, belonging to the X family of DNA polymerases, performs most of the gap-filling synthesis in the course of short-patch BER in nuclei [173,174] and in mitochondria [175,176]. In addition to the polymerase activity, Pol β also possesses a 5′-dRP lyase activity [177]. In addition, Pol β is involved in long-patch BER [178,179,180].

**Figure 3:**
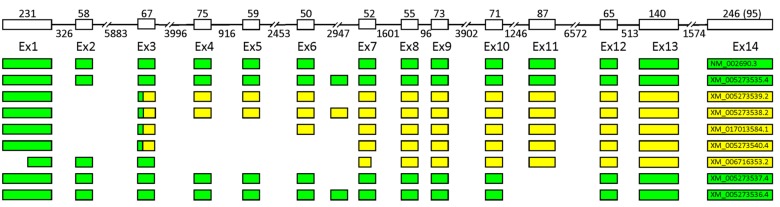
The schematic intron–exon organization of the human *POLB* gene and its common alternative splicing products. Untranslated regions are yellow, coding sequences are green.

Pol β is encoded by a single-copy 34 kb gene located on chromosome 8p11 [181]. The *POLB* gene consists of 14 exons ranging in size from 50 to 233 bp and 13 introns (Figure 3) [173,181,182,183]. The major Pol β isoform is a 39 kDa protein consisting of 335 amino acid residues. Pol β is folded into two distinct domains, each associated with a specific functional activity: the N-terminal 8 kDa domain (encoded by exons 1–4) shows the dRP lyase activity and the C-terminal 31 kDa domain (encoded by exons 5–14) possesses the DNA-polymerase activity [184,185].

*POLB* is a housekeeping gene expressed at low levels throughout the cell cycle [186]. Remarkably, the *POLB* gene is highly conserved in mammals (e.g., 99% among primates and 87% between rat and human), but the frequency of *POLB* splice variants in human cells is extremely high, reaching half total *POLB* transcripts [181,187,188,189,190,191,192,193,194]. About 60 splice variants of *POLB* were detected by sequencing of mRNAs and RT-PCR in different types of human normal tissues and cancer cells (Supplementary Materials). Much lower *POLB* splice variants levels were reported in other mammalian and non-mammalian species [188,191,194], suggesting that the *POLB* splice variants pattern observed in humans is not evolutionarily preserved and might play some adaptive functions. The majority of the *POLB* splice variants contain a premature terminating codon and are not able to produce a functional protein (Supplementary Materials).

Common *POLB* splice variants include deletions of exons 2, 4, 5, 6, 11, and 13 (∆), separately and in various combinations (Figure 3). Deletion of exon 2 (∆2) is the most frequent alternative splice event found almost in all cell types and tissues studied and conserved among primates [188,192]. Deletion of exon 2 leads to a frameshift and premature transcription termination in exon 3, resulting in production of truncated protein containing the first 26 amino acid residues [187,190]. The translated protein of 29 kDa should possess single-strand DNA binding and dRP-lyase activities. However, overexpression of this splice isoform does not rescue hypersensitivity to the cytotoxic effect of methyl methanesulfonate (MMS) in Pol β null cells and does not affect alkylating agent sensitivity and BER capability of Pol β proficient cells [192]. It was shown that the Ex2Δ mRNA is not translated in vitro [191] and its translation product is non-detectable in cell extracts (even in the presence of proteasome inhibitors), suggesting that deletion of exon 2 gives rise to a non-coding RNA transcript that could either represent unproductive and nonfunctional splice variant of *POLB* or modulate target mRNAs [192]. The possible role of Ex2Δ mRNA as a post-transcriptional regulator is supported by its localization at the sites of the active translation on polyribosomes, polyadenylation, and longer half-life [192]. A two-fold increase in the Ex2Δ *POLB* expression level does not induce any change in the levels of *POLB* mRNA but other genes could possibly be regulated.

The only confirmed protein isoform of Pol β detected in cells by Western-blot, along with the wild type Pol β, is a 36 kDa protein derived from translation of Ex11Δ mRNA [195,196,197]. Exon 11 encodes amino acid residues 208–236 and its deletion leads to the loss of 29 amino acids from the palm domain of Pol β. In many studies, the Ex11Δ variant was observed in tumors but not in the corresponding normal tissues [188,195,196,197,198,199,200,201], while no correlation with a gastric cancer phenotype was observed [192]. Cells expressing the Ex11Δ variant demonstrate decreased survival following exposure to alkylating agents [201,202,203]. The Ex11Δ protein suppresses BER reactions in transfected cells and cell extracts suggesting that this isoform acts as a dominant negative regulator of Pol β-dependent BER [201,202,204]. Expression of the Ex11Δ variant in mammary glands promotes carcinogenesis in transgenic mice after N-methyl-N-nitrosourea (MNU) treatment [203]. The Ex11Δ variant is capable of DNA gap-filling synthesis almost as effective as wild-type Pol β, and it binds a number of BER proteins: XRCC1, PARP1, APEX1, and TAF1D [204]. It is remarkable that binary complex of the Ex11Δ isoform and XRCC1 cannot perform the gap-filling reaction and has enhanced affinity to gapped DNA, suggesting that the Ex11Δ-Pol β-XRCC1 inhibits Pol β by substrate competition [204].

Deletion of several exons starting from exon 11 (∆11–13) are also common in tumors and cancer cell lines [188,190,191,198,200,205,206] and is possibly caused by mutations in the exon–intron junction [205]. HeLa cells transfected with the Ex11-13∆ variant express protein of 26.5 kDa lacking amino acids 208–304 of the catalytic core of Pol β, and display high sensitivity to alkylating agents and moderate sensitivity to UV and H_2_O_2_ [206]. This isoform is likely deficient in the DNA polymerase activity, [207] but retains the dRP lyase and DNA binding activities [208], and could act as a dominant negative mutant of Pol β. *POLB* splice variant Ex4–6,11–13∆ is associated with ovarian cancer [205].

Other common splice variants are retention of introns 6, 9, and 11 (Σ) [188,189,190,191,192,193,196,199,207,209]. Introns 6 and 9 were named exons α and β, respectively [187,191]. Retention of intron 6 does not affect the *POLB* reading frame and could possibly lead to production of a 42 kDa protein containing an additional 35 residues in the α-spiral region of the fingers domain [187]. The Σ exon α isoform produced in *E. coli* retains the catalytic activity but demonstrates reduced solubility [187]. There is no evidence of the existence of this isoform at the protein level in human cells yet.

Interestingly, it was observed that suppression of the nonsense-mediated decay pathway in human fibroblasts increases the splice variant frequency and amount of *POLB* isoforms containing a premature stop codon [190]. Therefore, part of the unproductive *POLB* splice variants could play a role in the nonsense-mediated mRNA decay mechanism.

## 4. Conclusions

Transcripts encoding BER enzymes undergo extensive alternative splicing that serves diverse purposes. It leads to production of proteins with grossly different catalytic capacity compared with their major annotated isoform (as OGG1-1 and -2), regulates their intracellular transport (as in UNG1 and UNG2), or provides a post-transcriptional regulation mechanism. Such regulation can include production of truncated proteins that act as dominant negative factors and inhibit the activity of the major isoform by substrate competition (e.g., Pol β Ex11Δ isoform), production of non-coding RNAs modulating target mRNAs (e.g., Pol β Ex2Δ isoform), or production of non-functional RNA isoforms degraded by nonsense-mediated decay. At the same time, the functions of many alternative splice variants of BER proteins remain largely unexplored. 

In some cases, splicing-affecting mutations in BER genes have been directly shown to be pathogenic. This is best illustrated by the example of *MUTYH*, where a number of splicing-affecting mutations, both germline and somatic, have been discovered in human tumors. Although their frequency is apparently lower compared to the well-characterized missense and truncating *MUTYH* mutations causative of colorectal polyposis and cancer, there is also a growing number of observations that aberrantly spliced transcripts can be produced even in the absence of mutations through splicing dysregulation in cancer cells [210,211]. Presently, the research field of normal and pathogenic splicing in base excision repair is ripe for discovery.

## Figures and Tables

**Table 1:** Experimentally confirmed and predicted mRNA and protein isoforms of major BER genes.

Gene	mRNA Experimentally Confirmed/Additionally Predicted *	Polypeptides Translated from Experimentally Confirmed/Additionally Predicted mRNA
*UNG*	2/0	2/0
*TDG*	2/1	2/1
*SMUG1*	32/8	5/0
*MBD4*	5/1	5/1
*NTHL1*	3/1	3/1
*MUTYH*	13/23	9/8
*OGG1*	13/7	13/6
*MPG*	3/1	3/0
*NEIL1*	4/16	4/8
*NEIL2*	8/0	3/0
*NEIL3*	1/1	1/1
*APEX1*	4/0	1/0
*POLB*	60/16	2/2

* Additionally predicted from the reference human genomic sequence (GRCh38.p13 Primary Assembly) by Gnomon, a module of the NCBI Genome Assembly and Annotation Pipeline [57].

**Table 2:** Pathogenic splice variants of *MBD4*, *NTHL1*, *OGG1*, *NEIL1*, and *NEIL2* genes.

HGMD Accession	Genomic Coordinates and Human Genome Variation Nomenclature	Variant Class	Phenotype	Reference
MBD4				
CS187177	c.335+1G>A	DM?	Glioblastoma	[67]
CS187176	c.1562-1G>T	DM?	Uveal melanoma	[67]
NTHL1				
CS1512540	c.709+1G>A	DM	Nth endonuclease III-like 1 deficiency	[69]
OGG1				
CM024572	c.137G>A	DM	Colorectal cancer	[70]
CS1515648	c.898+2T>G	DP	Breast cancer, in women, association with	[71]
CS1515649	c.948+2T>G	DP	Breast cancer, in women, association with	[71]
NEIL1				
CS088022	c.434+2T>C	DFP	Altered splicing	[72]
NEIL2				
CS053476	c.492-8C>T	DM?	Multiple colorectal adenoma	[73]

HGMD—Human Gene Mutation Database; DM—disease-causing mutation; DM?—probable disease-causing mutation; FP—in vitro or in vivo functional polymorphism; DP—disease-associated polymorphism; DFP—disease-associated polymorphism with additional functional evidence.

**Table 3:** Pathogenic splice variants of *MUTYH* gene.

HGMD Accession	Genomic Coordinates and HGVS Nomenclature	Variant Class	Phenotype	Reference
CS031780	c.389-1G>A	DM	Colorectal cancer	[130]
CS042821	c.389-1G>C	DM	MUTYH-associated polyposis	[131]
CS150026	c.462G>A	DM	MUTYH-associated polyposis	[132]
CS065596	c.463-1G>C	DM	MUTYH-associated polyposis	[133]
CS1717140	c.504+2T>C	DM	Cancer	[134]
CS1620236	c.577-2A>G	DM	Ovarian carcinoma	[135]
CS083952	c.690G>A	DM	Multiple colorectal adenomas	[72]
CS072232	c.691-1G>A	DM	Adenomatous polyposis coli, attenuated	[136]
CS150027	c.788+3A>G	DM	MUTYH-associated polyposis	[132]
CS031781	c.933+3A>C	DM	Colorectal cancer	[130]
CS042822	c.934-2A>G	DM	Gastric cancer	[128]
CS107266	c.998-13T>G	DM	Colon cancer	[137]
CS077659	c.998-1G>T	DM	Adenomatous polyposis coli	[129]
CS077658	c.1038G>A	DM	Adenomatous polyposis coli	[129]
CS1717138	c.1186+1G>A	DM	Cancer	[134]
CS1723999	c.1186+2T>C	DM	Breast cancer	[138]
CS050108	c.1187-2A>G	DM	Colorectal cancer	[139]
CS065595	c.1518+2T>C	DM	MUTYH-associated polyposis	[133]
CS083951	c. Not yet available	DM	Adenomatous polyposis coli	[140]
CS024315	c. Not yet available	FP	Reduced translation efficiency	[120]
CS171407	c.348+20G>A	DM?	MUTYH-associated polyposis	[141]
CS171414	c.388+56G>A	DM?	MUTYH-associated polyposis	[141]
CS171412	c.690+21C>A	DM?	MUTYH-associated polyposis	[141]
CS171415	c.997+5G>A	DM?	MUTYH-associated polyposis	[141]
CS171411	c.1187-27C>T	DM?	MUTYH-associated polyposis	[141]
CS171409	c.1477-28G>A	DM?	MUTYH-associated polyposis	[141]
CS1711315	c.1477-17C>G	DM?	Susceptibility to colorectal cancer	[142]
CS1711316	c.1519-14C>G	DM?	Susceptibility to colorectal cancer	[142]

DM—disease causing mutation; DM?—probable disease causing mutation; FP—in vitro or in vivo functional polymorphism.

## Supplementary Materials

Supplementary materials can be found at https://www.mdpi.com/1422-0067/20/13/3279/s1.

## Abbreviations

BER	base excision repair
AP	an abasic site
APEX1	AP endonuclease 1
dRP	5′ deoxyribose phosphate moiety
Pol β	DNA polymerase beta
UNG	uracil–DNA glycosylase
TDG	thymine–DNA glycosylase
SMUG1	single-strand-selective monofunctional uracil-DNA glycosylase 1
MBD4	methyl-binding domain-containing protein 4
NTHL1	endonuclease III-like protein
OGG1	8-oxoguanine–DNA glycosylase
MUTYH	MutY homolog
MPG	methylpurine–DNA glycosylase
NEIL1, NEIL2, NEIL3	endonuclease VIII-like proteins
PCNA	proliferating cell nuclear antigen
RPA	replication protein A
MTS	mitochondrial targeting sequence
NLS	nuclear localization signal
MMS	methyl methanesulfonate
