# Supplementary material for: Isoforms of Base Excision Repair Enzymes Produced by Alternative Splicing
Source: Int J Mol Sci. 2019 Jul 3;20(13):3279. doi: 10.3390/ijms20133279 (PMC6651865; doi:10.3390/ijms20133279)
Supplement: Supplementary file 1 [file ijms-20-03279-s001.pdf]

Review

Isoforms of base excision repair enzymes produced by alternative splicing

Elizaveta O. Boldinova[1], Rafil Khairullin[2], Alena V. Makarova[1,]* and Dmitry O. Zharkov[3,4,]*

[1]RAS Institute of Molecular Genetics, 2 Kurchatova Sq., Moscow 123182, Russia
[2]Institute of Fundamental Medicine and Biology, Kazan (Volga Region), Federal University, 420012 K.Marx, 18 Kazan, Russia
[3]Novosibirsk State University, 1 Pirogova St., Novosibirsk 630090, Russia
[4]SB RAS Institute of Chemical Biology and Fundamental Medicine, 8 Lavrentieva Ave., Novosibirsk 630090, Russia

***** Correspondence: amakarova-img@yandex.ru; Tel. +7-499-1960015 (A.V.M.) and dzharkov@niboch.nsc.ru; Tel. +7-383-3635187 (D.O.Z.)

Supplementary table. Alternative splice isoforms of *POLB* gene

Alternative splice isoforms	Premature termination codon	Frameshift	Protein	kDa	References
∑exα (in6)	-	-	370	42	8–10,17, 18
∑exβ (in9)	+	+	193		10,17,18
∑exα,exβ	+	+	228		10,17,18
∑exβ, part in3	ND	ND	96		18
∑in11	+	+	350		13,15,19
∑exα, Δex2	+	+	26		8,16
∑exβ, Δex2	+	+	26		10,18
∑19bp, Δex2	+	+	26		8
∑exβ, Δ2,4-6	+	+	26		10
∑exα, Δex11,13	-	-	294		3,5
Δpart ex1-8	+	+	182		3
Δex2	+	+	26		5,7–10,13,15–19,21
Δex2,3	+	+	28		10, 17
Δex2,3,11	+	+	28		10,17,18
Δex2,4	+	+	26		19
Δex2,4,5	+	+	26		8,10,18
Δex2,4-6	+	+	26		8,10,19
Δex2,4,5,9	+	+	26		19
Δex2,part ex 4, ex11	+	+	26		19
Δex2,4-13	+	+	26		19
Δex2,4,5,11	+	+	26		10
Δex2,4-6,11	+	+	26		10,16
Δex2,4-6,12,13	+	+	26		10
Δex2,4-11	+	+	26		16
Δex2,5	+	+	26		18
Δex2,5,6,9	+	+	26		19
Δex2-5,13	-	-	164		18
Δex2-6	ND	ND	42		18,19

Δex2,7,8,9	+	+	26		19
Δex2,9	+	+	26		16,19
Δex2-9	ND	ND	44		18
Δex2,11	+	+	26		10,16,17,18
Δex2,11-13	+	+	26		10,17, 18
Δex2,12,13	+	+	26		10,18
Δex2,13	+	+	26		18
Δex2-13	-	-	51		10,17, 19
Δex3-5	ND	ND	268		18
Δex3-6,10,11	ND	ND	44		10
Δex3-6,11-13	ND	ND	44		10
Δex3-6,9,11	ND	ND	46		16
Δex3-9,12,13	ND	ND	45		10
Δex4	-	+	310		10,17,18,19
Δex4,5	+	+	96		8,10,17,19
Δex4-6	+	+	70		8, 19
Δex4,5,12,13	+	+	71		18
Δex4-6,11-13	+	+	71		11
Δex4-9,11-13	ND	ND	118		5
Δex4-10	-	-	190		10,17,18
Δex4-11	-	-	161		10,17,18
Δex4-11,13	-	-	93		10, 17
Δex4-13	-	-	93		10,17, 18
Δex7,8	ND	ND	128		19
Δpart ex10-part ex13	ND	ND	ND		20
Δex11	-	-	306	36	1–6,9,10,13–23
Δpart ex 11	ND	ND	ND		20
Δex11-13	-	+	261	26,5	10,12,17,18,21
Δex12,13	-	-	290		10,17,18
Δ part ex12-part ex 13	ND	ND	ND		20
Δex13	-	-	267		18
Δex14	-	-	298		5

ND – no data

References

1. Bhattacharyya, N.; Banerjee, S. A variant of DNA polymerase b acts as a dominant negative mutant. *Proc Natl Acad Sci U S A*. 1997, 94(19), 10324–10329.
2. Bhattacharyya, N.; Chen, H.-C.; Grundfest-Broniatowski, S.; Banerjee, S. Alteration of hMSH2 and DNA Polymerase b Genes in Breast Carcinomas and Fibroadenomas. *Biochemical and Biophysical Research Communications*. 1999, 259(2), 429 – 435.
3. Bhattacharyya, N.; Chen, H.-C.; Comhair, S.; Erzurum, S.; Banerjee, S. Variant Forms of DNA Polymerase β in Primary Lung Carcinomas. *Dna Cell Biol.* 1999, 18, 549 – 554.
4. Bhattacharyya, N.; Banerjee, S. A Novel Role of XRCC1 in the Functions of a DNA Polymerase β Variant. *Biochemistry*. 2001, 40(30), 9005 – 9013.

5. Bhattacharyya, N.; Chen, H.-C.; Wang, L.; Banerjee, S. Heterogeneity in Expression of DNA Polymerase β and DNA Repair Activity in Human Tumor Cell Lines. *Gene Expr*. 2002, 10(3), 115 – 123.
6. Bhattacharyya, N.; Banerjee,T.; Patel, U.; Banerjee, S. Impaired repair activity of a truncated DNA polymerase β protein. *Life Sci*. 2001, 69(3), 271 – 280.
7. Chyan Y.-J.; Ackerman, S.; Shepherd, N. S.; McBride, O. W.; Widen, S. G.; Wilson, S. H.; Wood, T.G. The human DNA polymerase B gene structure. Evidence of alternative splicing in gene expression. *Nucleic Acids Res*.1994, 22(14), 2719 – 2725.
8. Chyan Y.-J.; Strauss, P.R.; Wood, T.G.; Wilson, S.H. Identification of Novel mRNA Isoforms for Human DNA Polymerase ß. *DNA Cell Biol*. 1996, 8, 653 – 659.
9. Chen H.-C.; Bhattacharyya, N.; Wang, L.; Recupero, A. J.; Klein, E. A.; Harter, M. L.; Banerjee, S. Defective DNA repair genes in a primary culture of human renal cell carcinoma. *J Cancer Res Clin Oncol*. 2000, 126(4), 185 – 190.
10. Disher. K.; Skandalis, A. Evidence of the modulation of mRNA splicing fidelity in humans by oxidative stress and p53. *Genome*. 2007, 50(10), 946 – 953.
11. Kalyani, K.; Bhattacharya, C.; Bhattacharyya, N. Association of a Newly Identified Variant of DNA Polymerase Beta (polβΔ63-123, 208-304) with the Risk Factor of Ovarian Carcinoma in India. *Asian Pac J Cancer Prev*. 2012, 13(5), 1999 – 2002.
12. Kalyani, K.; Chakraborty, A.; Bhattacharyya, N. HeLa Cells Containing a Truncated Form of DNA Polymerase Beta are More Sensitized to Alkylating Agents than to Agents Inducing Oxidative Stress. *Asian Pac J Cancer Prev.* 2015, 16(18), 8177 – 8186.
13. Nowak, R.; Bieganowski, P.; Konopinski, R.; Siedlecki, J. A. Alternative Splicing of DNA Polymerase β mRNA is not Tumor-Specific. *Int J Cancer.* 1996, 68(2), 199 – 202.
14. Kakali, P.; Kalyani, K.; Bhattacharyya, N. Identification of an Endoplasmic Reticulum Membrane Protein Interacting with DNA Polymerase Beta by a Yeast Two-Hybrid Screen. *Z Naturforsch C.* 2014, 69(1-2), 81 – 88.
15. Sadakane, Y.; Maeda, K.; Kuroda, Y.; Hori, K. Identification of mutations in DNA polymerase β mRNAs from patients with Werner syndrome. *Biochem Biophys Res Commun*. 1994, 200(1), 219 – 225.
16. Simonelli, V.; D'Errico, M.; Palli, D.; Prasad, R.; Wilson, S.H.; Dogliotti E. Characterization of DNA polymerase β splicing variants in gastric cancer: the most frequent exon 2-deleted isoform is a non-coding RNA. *Mutat Res.* 2009, 670(1-2), 79 – 87.
17. Skandalis, A.; Uribe E. A survey of splice variants of the human hypoxanthinephosphoribosyl transferase and DNA polymerase beta genes: products of alternative or aberrant splicing? *Nucleic Acids Res.* 2004, 32(22), 6557 – 6564.
18. Skandalis, A.; Frampton, M.; Seger, J.; Richards M.H. The adaptive significance of unproductive alternative splicing in primates. *RNA*. 2010, 16(10), 2014 – 2022.
19. Thompson, T.E.; Rogan, P.K.; Risinger, J. I.; Taylor J. A. Splice Variants but not Mutations of DNA Polymerase β Are Common in Bladder Cancer. *Cancer Res*. 2002, 62(11), 3251 – 3256.
20. Wang, L.; Patel, U.; Ghosh, L.; Banerjee, S. DNA Polymerase β Mutations in Human Colorectal Cancer. *Cancer Res*. 1992, 52(17), 4824 – 4827.
21. Wang, L.; Banerjee, S. Mutations in DNA polymerase ß occur in breast, prostate and colorectal tumors. *Int J Oncol*. 1995, 6(2), 459 – 463.

22. Wang, L.; Bhattacharyya, N.; Chelsea, D. M.; Escobar, P. F.; Banerjee, S. A Novel Nuclear Protein, MGC5306 Interacts with DNA Polymerase β and Has a Potential Role in Cellular Phenotype. *Cancer Res.* 2004, 64(21), 7673 – 7677.
23. Wang, L.; Bhattacharyya, N.; Thangaiyan, R.; Wang, L.; Banerjee, S. Mammary carcinogenesis in transgenic mice expressing a dominant-negative mutant of DNA polymerase β in their mammary glands. *Carcinogenesis*. 2007, 28(6), 1356 – 1363.
